# Experiences of EU and non-EU internationally educated nurses and midwives in the UK: a scoping review

**DOI:** 10.1186/s12912-025-04080-y

**Published:** 2025-12-02

**Authors:** Daisy Mpando, Yingxi Zhao, Mike English, Attakrit Leckcivilize

**Affiliations:** 1 NDM Centre for Global Health Research, Nuffield Department of Medicine, University of Oxford, S Parks Rd, Oxford, OX1 3SY UK; 2 KEMRI-Wellcome Trust Research Programme, Nairobi, Kenya

**Keywords:** Migration, Overseas nurse, Integration, Bullying, NHS

## Abstract

**Background:**

The UK’s health and care system is increasingly dependent on international recruitment to fill workforce gaps. In 2022–2023, nearly half of new Nursing and Midwifery Council (NMC) registrants were internationally educated nurses and midwives (IENs), with most coming from non-EU countries such as India, the Philippines, and Nigeria. While European Union (EU) nurses played a substantial role in NHS recruitment during the early 2010s, Brexit and subsequent immigrant policy changes contributed to a decline in EU recruit and a shift toward non-EU sources. This shift highlights the importance of understanding the differing experiences of EU and non-EU IENs. This scoping review explores recent literature on IENs’ experiences in the UK, with attention to similarities and differences between these groups.

**Methods:**

We searched Ovid MEDLINE, Ovid Embase, Ovid PsycINFO, EBSCOhost CINAHL, and Web of Science for peer-reviewed articles that explored the experiences of IENs in the UK between 2010-2024. Three reviewers screened articles for eligibility, and data were charted and coded thematically.

**Results:**

Thirty-three studies met inclusion criteria. We identified eight key themes: (1) migration motivation, (2) registration processes, (3) workplace adaptation, (4) deskilling and recognition, (5) discrimination, (6) job satisfaction, (7) social integration, and (8) coping and support. IENs frequently migrated in search of better professional opportunities but encountered complex processes. Many struggled to adapt to the workplace, with non-EU IENs more likely to report deskilling, limited recognition of prior experience, and visa-related restrictions. Discrimination was a persistent issue, particularly for Black and non-EU IENs, leading to poor job satisfaction and career progression. EU nurses, while previously benefitting from automatic qualification recognition, increasingly reported marginalisation following Brexit. While some IENs reported receiving good support from their employers and managers, others described inconsistent or absent support, and many relied heavily on religion or informal peer networks, especially from shared cultural or national backgrounds.

**Conclusion:**

IENs in the UK face diverse barriers spanning regulation, professional adaptation, social integration, and discrimination. These challenges vary by region of origin, particularly between EU and non-EU IENs. As international recruitment continues, tailored and sustained action is needed to improve IENs’ integration, retention, and wellbeing.

**Supplementary Information:**

The online version contains supplementary material available at 10.1186/s12912-025-04080-y.

## Background

Like many high-income countries, the UK increasingly relies on overseas health and care workers to address long-standing staff shortages. One in five NHS or adult social care workers in England is recruited internationally [1, 2]. Half of the new joiners to the Nursing and Midwifery Council (NMC) register in 2022–2023 were internationally educated nurses and midwives (hereinafter ‘IENs’) [1]. The UK’s reliance of IENs is particularly higher than that of other key EU countries, for example Germany (9%) and Italy (5%) [2], and such reliance will likely persist at least until 2036 [3]. However, the government can only succeed in achieving its staffing goals if health and care employers can successfully integrate and retain the IENs they recruit, which is a question of growing importance.

A substantial body of literature, including systematic and scoping reviews, examines various aspects of IENs in the UK and globally, focusing on migration trends, motivations, recruitment, and adaptation experiences (see Additional file 1). For UK-specific reviews, Nichols and Campbell (2009) first reviewed studies from 1995 to 2007, highlighting IENs’ adaptation to British nursing, perceived deskilling, and racial discrimination [4]. Lin et al. (2018) examined studies from 2002 to 2017, similarly reporting unequal treatment of IENs [5]. Bond et al. (2020) focused on qualitative studies published between 2010 and 2019, specifically on non-EU IENs, and found ongoing challenges in professional and socio-cultural integration [6]. The most recent review by Omiyi et al. (2024) summarised the experiences of internationally educated healthcare workers in the UK, calling for more inclusive systems of support and better support for professional growth and wellbeing [7].

While earlier reviews captured patterns of inequality and exclusion, recent UK political and policy developments have likely shifted the experiences of IENs in important ways. While European Union (EU) nurses played a substantial role in NHS recruitment in the early 2010s, Brexit - officially enacted in January 2020 following the 2016 referendum - led to a sharp decline in EU nurse registrations with the NMC and made the UK a less attractive destination for EU migrants [8]. In response, the government launched a special category of skilled worker visa, the Health and Care Worker visa, in August 2020, which featured reduced visa fees and exemptions from immigration health surcharges. This visa route drove an initial surge in non-EU skilled worker visas in 2021, with IENs being a significant part of this increase. In 2022, the majority of IENs came from non-EU countries, including India (46% of sponsored nurses), the Philippines (22%), Nigeria (14%), Ghana (6%), and Zimbabwe (3%) [8].

These shifting recruitment patterns raise important questions about how the experiences of EU and non-EU IENs may differ in the post-Brexit, post-pandemic landscape. EU nurses may face challenges related to the loss of automatic recognition of qualifications and changes to freedom of movement, which previously allowed for easier migration and employment within the UK. In contrast, non-EU IENs may experience barriers related to visa requirements, cultural adaptation, racial discrimination, and professional integration. Understanding how these pathways shape workplace experiences is essential to developing effective support mechanisms for the UKs increasingly diverse nursing and midwifery workforce.

In this scoping review, we aim to summarise recent evidence on IENs’ experiences in the UK, with particular attention to differences between EU and non-EU nurses and midwives. While much of the existing literature was collected before or during the early phases of recent policy changes, our analysis considers how evolving recruitment patterns and regulatory shifts may shape or reframe the interpretation of past findings. This review seeks to inform UK policymakers, employers, professional associations, and community organisations in developing more responsive and inclusive policies to support the recruitment, integration, and retention of a diverse nursing and midwifery workforce. More broadly, this paper may also offer valuable insights for policymakers in other countries navigating major immigration policy changes and seeking to understand their impacts on the internationally educated health workforce.

## Methods

We followed the five steps of Arksey and O’Malley method [9] for scoping reviews. Our aim was to systematically map the existing literature on the experiences of IENs in the UK, with particular attention to the comparison between those from EU and non-EU countries. To guide the search strategy and ensure a broad range of literature was captured, our research question was: “What are the experiences of internationally educated nurses and midwives working in the UK, and how do these differ between those from EU and non-EU countries?” This scoping review has been registered on the Open Science Framework (https://osf.io/qk9hg), and our reporting follows the PRISMA-ScR checklist.

### Search strategy and screening

We conducted a systematic search using Ovid MEDLINE, Ovid Embase, Ovid PsycINFO, EBSCOhost CINAHL, and Web of Science to obtain relevant articles. These databases were selected to ensure comprehensive coverage of biomedical, nursing, psychological, and interdisciplinary health literature relevant to the experiences of IENs in the UK.

We included all types of study designs published between January 2010 and March 2025. We focused on this time period to capture the past 15 years of international recruitment trends, including the expansion and subsequent decline of EU nurse inflows post-Brexit, alongside the rise in non-EU recruitment. Search terms combined keywords related to internationally qualified nurses, migration, and the UK. To capture studies involving migration from both high-income and low- and middle-income countries, we used the Cochrane Effective Practice and Organisation of Care (EPOC) LMIC filter [10], as well as a list of eight high-income countries and territories identified in 2022 Nursing and Midwifery Council data as the most common source countries (Poland, Ireland, Romania, Australia, United States, United Arab Emirates, Saudi Arabia, and Hong Kong) [11]. The full search strategy is provided in Additional File 2.

The initial search was conducted in November 2023, covering publications from January 2010 to November 2023. An updated search was conducted in March 2025 to identify studies published between November 2023 and March 2025. We focused primarily on English-language studies.

After deduplication, citations were imported into Rayyan for title and abstract screening. Studies were included if their primary focus was the experiences of IENs within the UK. We also included papers that studied a broader group of health professionals but provided separate data on nurses or midwives. We excluded studies focusing primarily on pre-arrival migration experiences, as well as policy briefs, opinion pieces, and review articles. Our inclusion and exclusion criteria are further detailed in our OSF registry.

**Fig. 1:**
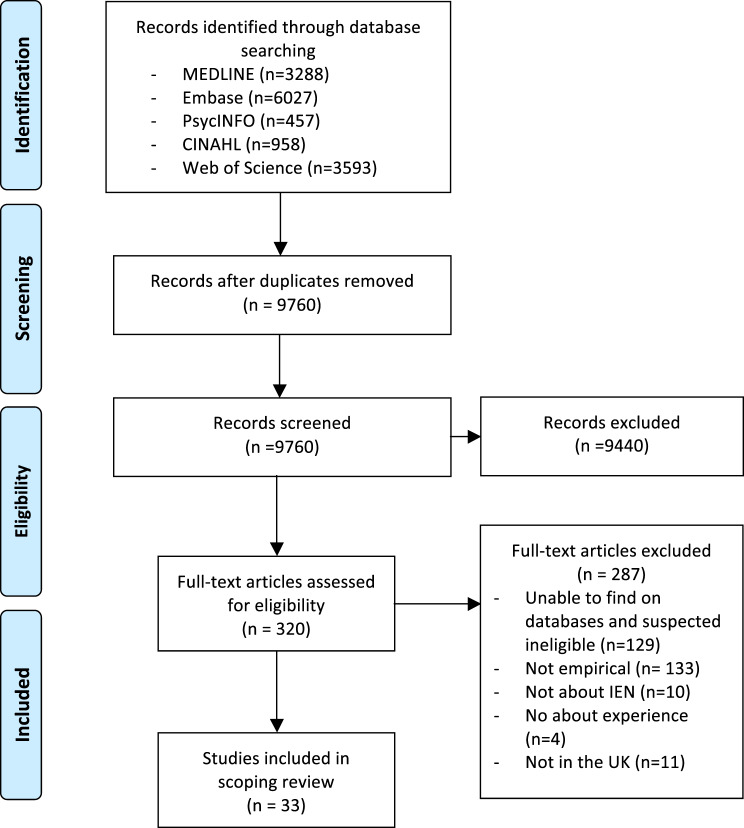
PRISMA diagram

For the initial screening, DM reviewed all titles and abstracts to assess eligibility for full-text review, with a random subset (40%) independently reviewed by a second reviewer (YZ or AL). All full-text articles were reviewed by DM and a second reviewer (YZ or AL). For the updated search, YZ screened all titles, abstracts, and full texts, with discussions held with DM and AL. Disagreements at any stage were resolved through discussion among the three reviewers. The PRISMA flow diagram is shown in Fig. 1.

### Data charting, collation and reporting

Data from each included study were extracted by DM and YZ using a standardised Microsoft Excel template, capturing: authors, year of publication, study aim, design, setting (NHS or care home, specific region), population characteristics (e.g. country of origin, professional role, time in UK), and key findings related to experiences. The included articles were then imported into NVivo (version 12) for coding. DM conducted initial inductive coding, which was then refined in discussion with YZ and AL. Final codes and the eight themes were reviewed and agreed upon by YZ. Although formal quality appraisal was not conducted, this aligns with guidance for scoping reviews [9], and is acknowledged as a limitation in the discussion.

We conducted comparative analysis to explore similarities and differences in experiences reported by EU and non-EU IENs, both within and across studies. Although only a small number of studies explicitly compared EU and non-EU IENs, we adopted a broad and inclusive approach. We analysed all UK-based studies focused on IENs, regardless of whether they included direct comparisons, and synthesised findings across studies to identify patterns relating to national origin or country of training. This allowed us to explore implied or inferred differences and draw comparative insights not made explicit in the original studies. We also examined whether any regional differences were reported in the studies (e.g., between different UK geographies).

## Results

Figure 1 summarises the result of the review process. Of 9,760 citations identified after deduplication, 33 met the inclusion criteria after the full-text review. The characteristics of the included studies are provided in Table 1. Twelve studies were published after 2020. One study explicitly included midwives in their study participants, whereas the rest all focused on nurses.

**Table 1:** Characteristics of included studies

Reference	Main study objectives	Year of publication	Study design and data collection	Study setting	Number of IENs participants	Country and territory of origin
**Studies that focus on EU IENs**						
[12]	To investigate education, job, and professional development satisfaction among intensive care unit nurses including nurses of various national background	2025	Quantitative (interview-based survey with open ended questions)	Secondary care NHS hospitals in Guilford and Papworth Everard	125	Poland, Portugal, Spain and Italy
[13]	To explore the reasons for Italian midwives’ decision to migrate, and their lived professional and emotional experiences	2023	Qualitative (interviews)	Multiple settings in the UK and other European countries	4	Italy
[14]	To use migration infrastructure to explain how Brexit and the COVID-19 pandemic affected the international flow of health workers to and from the NHS	2022	Mixed methods (secondary data analysis, interviews)	Secondary care NHS hospitals	1	Netherlands
[15]	To explore the experiences of EU and non-EU nurses post Brexit in the UK	2023	Qualitative (interviews)	Secondary care NHS hospitals in Midlands and Southwest of England	11	Philippines, Greece, Spain, Italy
[16]	To understand and describe the experiences and perceptions of migrant Spanish nurses working in the UK	2020	Quantitative (online survey with open ended questions)	Not clearly reported	371	Spain
[17]	To analyse the experience of Portuguese nurses working in the English NHS considering the individual and organizational factors that affect the quality and duration of nurses’ migration experience, future career plans and expectations	2020	Qualitative (interviews)	Secondary care NHS hospitals	11	Portugal
[18]	To advance knowledge in the context of human rights conflicts and ethical implications of the decision-making process of nurses who migrate between developed countries, such as from Italy to the United Kingdom, during times of recession	2017	Qualitative (open ended interviews)	Not clearly reported	26	Italy
**Studies that focus on non-EU IENs**						
[19]	To explore lived experiences of IENs with Nigerian heritage career progression on integration into UK healthcare services	2024	Qualitative (interviews)	Multiple settings in London	Not reported	Nigeria
[20]	To examine how female Nigerian doctors and nurses cope with racism and gendered racism they experience in their everyday working lives	2024	Qualitative (interviews)	Multiple settings in the NHS	12	Nigeria
[21]	To understand how African nurses use religious and spiritual coping to deal with racism in the UK workplace	2024	Qualitative (interviews)	Multiple settings in the NHS	43	8 African countries, e.g. Kenya, Nigeria, Malawi, Zimbabwe
[22]	To analyse the experiences of female Nigerian doctors and nurses working in the NHS	2022	Qualitative (interviews)	Multiple settings in the NHS	12	Nigeria
[23]	To understand Filipino nurses’ experiences of the ‘Test of Competence’ process, alongside the additional competency requirements of their sponsor Trust	2021	Qualitative (focus group discussions)	Secondary care NHS hospitals	21	Philippines
[24]	To analyse the migratory rationales and choices of Filipino nurses either about to embark or already working in the UK NHS	2021	Qualitative (focus group discussions)	Secondary care NHS hospitals	24	Philippines
[25]	To describe the experiences of nurses recruited from India who participated in an Overseas Nurses Program	2017	Qualitative (semi-structured interviews)	Secondary care NHS hospitals	16	India
[26]	To identify perceived barriers to UK nurse registration as experienced by internationally educated nurses working as healthcare assistants in the UK	2016	Qualitative (focus group discussions)	Secondary care NHS hospitals in London	11	Philippines, Nepal
[27]	To examine Nepali migrant nurses’ professional life in the UK	2015	Qualitative (in-depth interviews)	Multiple settings including secondary care NHS hospitals and care sector	21	Nepal
[28]	To explore how Jordanian nurses experienced the transition from home to host country to illuminate the elements of transformation	2015	Qualitative (semi-structured interviews)	Multiple settings	25	Jordan
[29]	To explore the links that South African-trained health workers who now live and work in the United Kingdom maintain with their country of training and the future migration plans of interviewees and the factors influencing those decisions.	2015	Qualitative (semi-structured interviews)	Not clearly reported	10	South Africa
[30]	To examine how overseas nurses encountered and overcame the challenges linked to recruitment and migration restrictions	2013	Qualitative (interviews)	Not clearly reported	34	Nepal, Malawi
[31]	To determine the experiences of South African trained health workers who have migrated to the United Kingdom	2014	Qualitative (interviews)	Not clearly reported	10	South Africa
[32]	To explore the experiences of racial microaggression among migrant nurses in the United Kingdom	2014	Qualitative (diary writing)	Secondary care NHS hospitals in midlands of England	11	Philippines, Kenya, Zimbabwe, Zambia
[33]	To gain an understanding of IENs’ experiences of working in the NHS in England	2014	Qualitative (interviews, focus group discussions)	NHS district general hospital in England	36	Philippines, South Africa, Sub-Saharan Africa, Caribbean
[34]	To explore Black African nurses’ experiences of equal opportunities, racism, and discrimination in four NHS trusts in north-eastern England	2013	Qualitative (semi-structured interviews, focus group discussions)	Secondary care NHS hospitals in north-eastern England	30	9 African countries including Malawi, Kenya, Ghana, Nigeria
[35]	To explore the perceptions and work experiences of internationally recruited neonatal nurses	2011	Qualitative (in-depth open-ended interviews)	Secondary care NHS hospitals in London	13	Jamaica and the Philippines
[36]	To explore barriers to effective and non-discriminatory practice when mentoring overseas nurses within the National Health Service (NHS) and the care home sector	2010	Qualitative (in-depth interviews)	Multiple settings including secondary care NHS hospitals and care sector	93	South Africa, Philippines, Ghana, India
[37]	To describe the experiences of nurses recruited from Ghana and the Philippines by a London NHS Trust	2010	Qualitative (focus group discussions)	Secondary care NHS hospitals in London	13	Ghana, Philippines
[38]	To explore the experience of and motivation of nurses who left Malawi to work in the UK	2010	Qualitative (interviews)	Multiple settings in London, Glasgow and Nottingham	7	Malawi, Nepal
**Studies that focus on mixed regions or did not report specific country groupings**						
[39]	To explore the migration motivations and experiences of initial integration for internationally recruited nurses	2024	Mixed methods (online survey with open ended questions)	Multiple settings in the NHS	655	Global, including India, Philippines, and other countries
[40]	To understand the experiences of internationally educated nurses’ first two years working and living in England	2024	Mixed methods (online survey with open ended questions)	Multiple settings in the NHS	773	Global, including India, Philippines, and other countries
[41]	To describe the English language required to deal with the daily demands of nursing in the UK and compare these abilities with the stipulated levels on the language test	2017	Qualitative (interviews)	Secondary care NHS hospitals in London	4	India, Philippines, Portugal, Hungary
[42]	To determine internationally registered nurses’ perception of discrimination, support, and their adjustment to a new environment in the NHS in England	2014	Quantitative (survey)	Secondary care NHS hospitals in England	188	21 countries e.g., Australia, China, Ghana, India, Kenya, South Africa
[43]	To explore internationally recruited neonatal nurses’ perceptions of their experiences of working in the NHS in London	2013	Qualitative (interviews)	Secondary care NHS hospitals in London	13	Not specified
[44]	To understand the recruitment and retention of overseas nurses	2012	Qualitative (in-depth interviews, observations)	Secondary care NHS hospitals in Northwest England	Not reported	India, Philippines, Spain

Seven studies focused on IENs from EU countries, 20 studies included or focused on those outside of the EU, and six studies examined mixed regions or did not report specific country groupings. For studies involving mixed regions, we explored differences in experiences between EU and non-EU IENs where possible. In addition, our synthesis compared findings from EU-specific and non-EU-specific studies to draw out inferred contrasts across studies where relevant.

In terms of study setting, 16 studies specifically investigated IENs’ experiences in secondary care NHS hospitals, and 11 focused on multiple settings or others. Twelve studies reported the geographic location of their setting in detail, including 7 that focused on London, and the rest covering Northwest of England, Midlands, etc. The majority of studies used a qualitative approach, except for two studies that used a quantitative survey approach and three that used a mixed-methods approach.

### Migration motivation and journey

Many studies documented the push and pull factors for IENs’ migration decisions before they arrive in the UK. These often include expectations for economic stability, career development opportunities and better social support systems and services 24, 38]. These positive pull factors often contrast sharply with the practical difficulties of securing employment in their countries of origins. For example, studies highlighted the impact of unemployment and lack of advancement opportunities in both EU countries such as Spain [16], Italy [13, 18], Portugal [17], and non-EU contexts such as Malawi [38] and South Africa [29]. In the Portuguese context, migration was also facilitated by the relatively low cost and ease of travel to the UK [17].

In addition to structural and professional factors, the influence of peers and social networks was consistently reported across EU and non-EU IEN studies. Word of mouth from friends already working in the UK, positive social media posts, and targeted recruitment campaigns were found to shape expectations and motivate migration. For instance, nurses from Portugal and Spain were influenced by advertisements and friends’ recommendations [16, 17], while similar dynamics were reported among Malawian and Filipino nurses, who were encouraged by peers and recruiter networks [30, 31, 38].

Some of these motivations also influenced IENs’ decisions to remain in the UK, even in the face of difficult or negative workplace experiences (shown in later themes). Retention was further shaped by family circumstances [29], fear of returning home without having achieved expected financial or educational gains [30, 44], and, in the case of EU nurses, uncertainty around future employment rights due to Brexit [16]. For some non-EU nurses, work in the UK was also seen as a stepping stone toward onward migration to countries like Australia or the United States [32].

### English language and competency test

Several studies, particularly those from non-EU countries described the lengthy process of passing the English language and clinical competency (OSCE) tests required for NMC registrations [23, 25, 26, 41]. Nurses from countries such as the Philippines and Ghana reported repeated failures in the IELTS exam, which was often viewed as unrelated to clinical practice and lacking in assessment of relevant soft skills, such as managing hierarchy or avoiding conflict with senior colleagues [26, 41]. The OSCE test was similarly described as a source of anxiety, particularly for non-EU IENs who faced the risk of being sent home if they failed initial adaptation programmes. As O’Brien and Ackroyd [44] noted, these nurses also faced further indignity by being paid as healthcare assistants - rather than as nurses - until they passed the required competency assessments.

EU nurses, by contrast, previously benefited from the automatic recognition of qualifications under EU law and were exempt from both English language and clinical competency testing [44]. However, post-Brexit changes to NMC policy now require most EU applicants to meet the same registration requirements as non-EU nurses - unless they qualify for exemptions such as recent practice in a majority English-speaking country or qualifications taught and examined in English [45]. While these changes have important implications, most studies in this review pre-date their full implementation and do not reflect recent developments such as the Supporting Information From Employers (SIFE) pathway. These issues are further explored in the discussion.

While earlier studies emphasised language proficiency testing as a major hurdle, more recent findings suggest a more nuanced picture of communication challenges in practice. In Pressley et al.’s survey of 773 nurses, 71.6% of international nurses reported no difficulties communicating with patients [40]. However, for those who did, the issues were often related to local accents, colloquialisms, and slang - rather than English proficiency per se. Nurses from the Philippines were significantly more likely to report communication difficulties with dialects and figurative speech than other countries [39], while similar concerns were raised by nurses from Ghana [37] and Italian midwives [13] qualitatively, who described the language barrier as “the main critical element” that limiting effective care delivery.

### Workplace adaption

Many IENs described significant challenges in adjusting to the British way of nursing, particularly during their early period of employment. Nurses from non-EU countries often expressed unfamiliarity or surprise with core aspects of UK nursing culture, such as family-centred care [35], the role of care assistants [34] and specific norms around communication. One Ghanaian nurse explained that in the UK, “speaking eye to eye with someone without even a blink is the British way of communication,” whereas in their home country, avoiding eye contact is considered a sign of respect [37]

Several studies described how British staff and managers sometimes perceived UK nursing norms as the only correct standard, framing differences in practice as “clinical mistakes” rather than recognising them as examples of international variation [36]. These perceptions often created tension and made it more difficult for IENs to feel accepted or understood within their new workplaces.

### Use of skills and career progression

Many non-EU IENs reported feeling underutilised in the UK, as their prior clinical experience and qualifications are not recognised [40]. Back in their home countries, they were often allowed to perform more advanced tasks such as drawing blood and applying intravenous (IV) cannulas [23, 28], whereas in the UK they were often given direct care and minor tasks [31, 43, 44]. IENs felt that being treated like students, was downgraded to the lowest nursing grade and “back to square one” [34, 43]. These frustrations were often intensified by exclusion from higher-status work [44] or being supervised by less-qualified colleagues [34]. In a few cases, supportive managers helped IENs leverage prior experience - such as one Nigerian nurse whose teaching background facilitated her career progression in the NHS [19]. In contrast, EU nurses – such as in studies from Spain and Portugal - were more likely to feel valued and recognised in their roles [16, 17].

This mismatch between expectations and reality was particularly common in the care home sector. Many non-EU nurses arrived in the UK through recruitment agencies that targeted private care employers, often without clearly explaining the nature of the work or employment rights [27, 38]. Nurses from countries such as Malawi and Nepal found these roles menial and repetitive, with limited clinical challenge or development. Yet many felt “trapped”, as their visa status was tied to their employer, limiting their ability to change jobs [27, 30, 38].

Access to career progression and development opportunities also varied. Many IENs described being overlooked for training or promotion, and eventually giving up on applying altogether [22, 32]. For those who viewed the UK as a temporary stepping stone to other countries such as Australia or the US, the lack of support for professional growth was demoralising and discouraged long-term retention [32].

### Bullying, racism and discrimination

IENs face multi-level discrimination, from their patients, their colleagues and senior managers as well as the organisation. Many Black IENs, particularly from non-EU countries e.g. Malawi and Ghana, described patients refusing care from them, often under the guise of concerns about English fluency [29, 32, 34, 35, 38]. Nurses were being mistaken for housekeepers or cleaners based on skin colour, or being questioned by police while on duty as a nurse manager [22]. One Black nurse reflected: “Because I am Black, I am going to be a housekeeper … When I say, I’m the nurse manager, they don’t understand that.” [22]

Discrimination from colleagues and managers was also common. Instances included the use of patronising and demeaning language, excessive criticism, and hostility stemming from the perception that they were “stealing local jobs” [32]. Some described being assigned only menial tasks due to assumptions about their abilities - for example, one UK-trained Malawian nurse was routinely sent to low-tech jobs because colleagues assumed, based on her ethnicity, that she could not handle specialised equipment [38]. Tensions were also reported with healthcare assistants, who at times refused instructions from IENs. One nurse stated, “Those on lower bands don’t respect you … because you are not British. When they work with seniors who are British, they do their jobs without being told” [39]. Despite there being official guidelines around bullying and discrimination, many IENs had to remain silent and not complain as they were under supervised practice [33, 36] or their work permit is linked with their visa [38]. Occasionally their grievances were “swept under the rug” from further escalation [32]. Amongst non-EU IENs, Black African nurses felt they were more discriminated against when compared with Indian, Pakistani and Filipino nurses [34, 42].

Additionally, EU nurses also reported instances of exclusion, particularly amid Brexit policy changes. One Dutch midwife recalled seeing a Facebook post from her manager stating “British jobs for British workers”, which deeply affected her [14]. Spanish nurses similarly described being treated as “second class” [16]. These highlighted how even EU nurses were not immune to perceived marginalisation.

### Job satisfaction

Job satisfaction among IENs was shaped by multiple factors, including perceived professional value, working conditions, and opportunities for development. Several studies reported high job satisfaction among EU nurses. For example, Polish nurses working in England reported significantly higher satisfaction with their education than their counterparts in Poland (4.44 vs. 3.88 out of 5), and slightly higher overall job satisfaction (3.82 vs. 3.18) [12]. Qualitative data suggested reasons include better professional development, work environments, and feelings of respect for their roles in the UK. Similarly, Italian midwives described high job satisfaction in the UK due to greater professional autonomy, flexible scheduling, and respectful team dynamics [13]. Spanish nurses also reported feeling welcomed and valued in the NHS, with opportunities for career progression such as training to become advanced nurse practitioners which do not exist in their home countries [16].

In contrast, job satisfaction was often lower among non-EU IENs. Even when they valued certain aspects of their roles, such as flexible hours or better patient care environments, these positives were sometimes overshadowed by the emotional toll of being deskilled, underused, or treated unequally compared to local staff, leading to diminished morale and disillusionment [30, 39, 43]. For example, while Indian and Jordanian nurses appreciated the greater autonomy in UK practice compared to their home countries, they were also disappointed when their clinical skills were not recognised or used in daily practice [25, 28].

### Social integration

Aside from professional and workforce integration, many IENs also faced challenges in integration culturally and socially. Feelings of isolation, homesickness, and culture shock were widely reported, often lasting well beyond the initial months of migration [17, 33, 44]. Jordanian nurses, for example, described difficulties socialising due to cultural norms around alcohol consumption [28], while others noted confusion over seemingly minor cultural differences such as dress codes or norms around eye contact [38].

Housing was a rising concern for more recent studies. Many IENs initially received employer-provided housing for a short period, but later faced difficulties securing private accommodation due to high costs, unfamiliar rental processes, and lack of guarantors [39]. These challenges led some to accept suboptimal housing far from their workplace, with 67% of 773 IEN surveyed by Pressley and colleagues reporting commuting difficulties [40].

Social integration issues were also reported for EU nurses especially in the post-Brexit era. Spanish and Greek nurses, despite being assumed to face fewer cultural barriers, described experiencing racism in public spaces - including discriminatory comments in pubs or being made to feel like outsiders despite their professional roles [15].

### Coping and support mechanism

Recent survey data suggest that many IENs feel supported by their employers: over 70% of respondents in one survey with 655 IENs reported receiving excellent support from their organisation [39]. Line managers, clinical educators, and mentors were particularly valued for bridging both personal and professional support needs. For example, studies highlighted that mentors were particularly effective in easing anxieties, building trust and supporting adaptation [25].

However, this level of support was not consistent across contexts. Many non-EU nurses, particularly those from Black African backgrounds, described the need to “tolerate the unacceptable” as part of their daily working lives [33, 43]. Compared to their Filipino, Indian, and Pakistani peers, they were less likely to feel supported by colleagues [42]. Organisational preparedness for supporting IENs varied widely. Some managers were unaware of the distinct learning needs of overseas nurses, or assumed they would adapt without tailored support - a concern noted particularly in relation to nurses from South Africa and the Philippines [26].

In the absence of formal structures, IENs often relied on personal resilience or faith as coping mechanisms. Religious beliefs were described as a source of strength, particularly when dealing with racism or exclusion [20, 21]. Peer support from colleagues from the same home countries also played a vital role for both EU and non-EU nurses. Nurses valued informal networks, buddy systems, and mentors from similar cultural or linguistic backgrounds, which helped mitigate feelings of isolation and anxiety. For example, Filipino and Indian nurses described drawing comfort from senior staff who shared their cultural background [23, 25]. Similarly, Portuguese nurses noted that migrating as a group or meeting other Portuguese staff on arrival helped ease their transition [17].

## Discussion

Our review summarised findings from 33 studies published between 2010 and 2024 on the experiences of IENs in the UK, with particular attention to similarities and differences between those from EU and non-EU countries. While migration motivations were broadly similar across both groups - often driven by better labour markets and professional development opportunities - experiences post-migration diverged in important ways. Non-EU nurses were more likely to report challenges with language and registration processes, professional deskilling, visa-related restrictions, and multi-level discrimination. EU nurses, by contrast, had previously benefited from the automatic recognition of qualifications under EU law, but more recent studies suggest growing experiences of marginalisation, particularly amid Brexit policy changes. Career progression challenges were also noted, with some IENs reporting limited access to advancement opportunities despite years of service.

Despite several major policy changes - including the introduction of the Health and Care Worker visa in 2020 [46], and recent revisions to Nursing and Midwifery Council (NMC) language requirements [45] - many of the longstanding challenges described in earlier reviews still persist [4, 5]. Brexit introduced new uncertainty for EU nurses, while non-EU nurses continue to face burdensome examination requirements and registration hurdles. Recent regulatory reforms, such as the Supporting Information From Employers (SIFE) pathway introduced in 2023 [47] and the tightening of visa sponsorship rules for care providers in 2024 [46], aim to improve fairness and oversight. However, most of the studies included in this review predate these specific changes. Ongoing evaluation will be needed to assess their actual impact. Of particular concern is the care home sector, where several studies highlighted that non-EU IENs were misinformed by recruitment agencies, employed in roles that underutilised their skills, and “trapped” by visa-tied contracts [27, 30, 38]. These concerns remain highly relevant, especially given recent policy changes in 2024 that restrict international recruitment to Care Quality Commission (CQC)-registered providers [48].

While only a small number of studies we reviewed explicitly compared EU and non-EU IENs [39–42, 44], our synthesis draws inferences across multiple country-specific studies to generate new insights. This comparative perspective, while not conclusive, enables the identification of broader patterns that may be missed in single-country analyses. It also signals the need for future research to more deliberately explore variations by region of origin, race, visa status, and registration route.

Regionally, there are some indications that the experiences of IENs also vary across the UK. Even in highly ethnically diverse cities such as London, IENs reported facing discrimination and racial stereotyping [22]. Other London-based studies pointed to intense work pressures and overstretched teams [33, 43]. A study from the Northeast England suggested that the relative scarcity of ethnic minorities may amplify the perceived threat of Black IENs, increasing their vulnerability to discrimination [34]. In the Midlands and Southwest, more subtle forms of exclusion were reported in less diverse workplace settings [15].

These findings align with several theoretical frameworks. Theories of informal and social closure [49, 50] help explain how IENs, particularly those from non-EU or Black African backgrounds, are often excluded from valued tasks, learning opportunities, or advancement pathways. O’Brien and Ackroyd’s (2012) work on assimilation and occupational closure similarly suggests that workplace norms and expectations can reinforce exclusion by treating British clinical standards as the only “correct” way of practising [44]. More broadly, Ryan’s differentiated embedding model [51] and the Home Office’s integration framework [52] underscores how migrants experience integration unevenly across multiple domains – e.g. work, housing, neighbourhood, and community life - and how this evolves over time. Together, these frameworks offer useful starting points for informing policies and interventions that aim to support IENs not just in the workplace, but across the broader spectrum in the community and society.

Our review also contributes to global debates on IEN migration. For instance, IENs in the US, Japan, and New Zealand report similar struggles with qualification recognition, cultural adaptation, and career stagnation [53–55]. These parallels suggest that the UKs IEN experience is not unique but part of a wider pattern shaped by how destination countries balance workforce needs with migration regulation and professional gatekeeping.

Our findings also speak to several implications for policy and practice. Given the ongoing and growing reliance on IENs across both the NHS and social care, more coordinated strategies are needed to support their recruitment, integration, and long-term retention. Promising initiatives such as the India English Language Programme [56] and the development of post-arrival induction guidelines modelled on those created for international medical graduates [57], should be considered, expanded and evaluated. Employers and trusts must invest in high-quality onboarding, cultural competence training, and equitable access to career development opportunities. Consistent, tailored mentorship programmes could help IENs feel more valued and supported, particularly during their early adjustment but also for longer term development. Notably, several studies also highlighted positive experiences: Italian midwives described high job satisfaction linked to greater autonomy and professional respect [13], while Spanish and Polish nurses noted feeling welcomed and appreciated within the NHS, particularly in relation to career progression and working conditions [12, 16]. These examples offer important insights into what supportive, inclusive environments can look like, and should be drawn upon to guide improvements across the health and care sector.

Several limitations should be considered while interpreting these findings. Firstly, this was a scoping review therefore we did not systematically assess the quality of the included articles. Second, only one reviewer ran the thematic analysis, though a second independent reviewer manually checked all primary codes and subthemes. Thirdly, most included studies did not report sufficient detail on nurses’ educational preparation or training in their countries of origin, limiting our ability to assess how this may influence their adaptation in the UK. Fourthly, while we aimed to capture the experiences of all IENs, including those from high-income countries, our search terms were limited to IENs, LMICs, and the eight high-income countries and territories most commonly identified in recent NMC data. Consequently, we might have missed studies not specifically labelled as such, including several that investigated the experiences of Black, Asian, and Mixed Ethnicity (BAME) nurses in the UK, which are potentially relevant. Finally, only a minority of studies differentiated experiences by region or type of NHS organisation, and more granular comparisons including by country and this remains a key area for future research.

## Conclusion

This review highlights both persistent and emerging challenges faced by IENs in the UK, with notable differences between those from EU and non-EU countries. These findings point to the need for more tailored and equitable policies that acknowledge the diverse pathways and barriers IENs encounter. In the context of ongoing workforce shortages and continued reliance on international recruitment, there is a pressing need for coordinated action by government, employers, professional bodies, and community organisations to better support IENs through the migration process, workplace and social integration, and long-term career development. This could include structured onboarding, clear progression pathways, accessible mentoring and peer support groups, and sustained investment in inclusive workplace cultures.

## Electronic supplementary material

Below is the link to the electronic supplementary material.

Supplementary Material 1

Supplementary Material 2

## Data Availability

All data relevant to the review are included in the article or uploaded as additional files.
