# Supplementary material for: Experiences of EU and non-EU internationally educated nurses and midwives in the UK: a scoping review
Source: BMC Nurs. 2025 Dec 2;24:1459. doi: 10.1186/s12912-025-04080-y (PMC12673689; doi:10.1186/s12912-025-04080-y)
Supplement: Supplementary file 1 — Supplementary Material 1 [file 12912_2025_4080_MOESM1_ESM.docx]

| **Article** | **Year searched** | **Country & setting** | **Main findings** |
| --- | --- | --- | --- |
| Chen G, Ogata Y, Sasaki M. Factors associated with burnout among internationally educated nurses: A scoping review. International Nursing Review. 2025 Mar;72(1):e13073. | Until Sept 2023 | Global | - Aimed to identify organisational and individual factors contributing to burnout among IENs - Poor working environments and high patient loads were key organisational contributors - Individual risk factors including younger age, limited local language proficiency, and short duration of employment |
| Omiyi D, Wilkinson E, Snaith B. Exploring the Motivations, Challenges, and Integration of Internationally Educated Healthcare Workers in the UK: A Scoping Review. Policy, Politics, & Nursing Practice. 2024 Nov 20:15271544241289605. | 2010-2023 | UK | - Aimed to explore the motivations, challenges, and integration experiences of internationally educated healthcare workers in the UK - Challenges include professional recognition, discrimination, language barriers, and socioeconomic factors affecting integration and retention - Highlighted the need for addressing recruitment practices, registration processes, and cultural competence training to create an inclusive environment for IEHWs - Supportive policies to enhance professional growth and well-being of IEHWs |
| Davis D, Sharma S, Taylor M. Language tests and tests of competence for registration purposes–a scoping review of the experiences of overseas nurses. Contemporary nurse. 2024 Jul 3;60(4):420-32. | Jan 2015 – Jan 2023 | UK | - Explored challenges faced by overseas-educated nurses undertaking the UK’s Nursing and Midwifery Council (NMC) registration tests - Competence/practice disparities, arbitrary failures, and financial implications associated with the Objective Structured Clinical Examination (OSCE) - Lack of robust research evaluating the effectiveness of these competency assessments |
| Thomas JB, Lee MA. Factors influencing the transition of foreign‐educated nurses to the US healthcare setting: A systematic review. International nursing review. 2024 Sep;71(3):440-56. | 2015-2022 | US | - Aimed to examine the impact of orientation programs on foreign-educated nurses’ transition to the US healthcare system - Quality orientation and organizational support improved job satisfaction and reduced turnover among these nurses - Key factors including peer support, workload, credentialing, communication, cultural adjustment, and coping strategies - a lack of research evaluating the effectiveness of orientation programs for foreign-educated nurses |
| Cubelo F, Parviainen A, Kohanová D. The impact of bridging education programs on internationally educated nurses becoming registered nurses in high‐income countries: A mixed‐methods systematic review. International Nursing Review. 2024 Aug 24. | Until April 2024 | High income countries | - Aimed to understand impact of bridging education programs for IENs from low- and middle-income countries seeking registration in high-income countries - Language proficiency as the critical factor influencing success in bridging programs - Tailored educational approaches to enhance nursing competence and address skepticism - Other cultural challenges during workplace transition, underscoring the importance of targeted support for continuous integration |
| Ubah CI, Goldspink S, Tsegay SM. Black African international nurses' experiences of pastoral support: A scoping review. International Journal of Nursing Studies Advances. 2024 Jun 1;6:100202. | Until Apr 2023 | UK | - Aimed to identify existing knowledge on pastoral support for IENs in the UK - Current research primarily consists of policy guidelines and expert opinions, lacking empirical research on IENs’ perspectives - Significant disparities in the support received by IENs, influenced by varying definitions and practices of pastoral care across UK healthcare institutions - Emphasized the need for research focusing on specific groups, such as Black African nurses, to understand their unique support needs and experiences |
| Njie-Mokonya N, Montoya L, Abebe N, Shorr R. Examining Workplace Practices Used to Facilitate Successful Integration of Internationally Educated Nurses Into Acute Care Settings: A Scoping Review. The Journal of Continuing Education in Nursing. 2024 Apr 1;55(4):195-201. | Jan 2012 and July 2022 | Acute care; global | - Aimed to examine workplace practices supporting the integration of IENs into acute care settings - Inconsistencies in integration practices across countries and institutions - Bridging programmes often failed to prepare IENs for real-world clinical demands - Tailored supervision and structured support within acute care environments to enable safe, full-scope practice |
| Ung DS, Goh YS, Poon RY, Lin YP, Seah B, Lopez V, Mikkonen K, Yong KK, Liaw SY. Global migration and factors influencing retention of Asian internationally educated nurses: a systematic review. Human Resources for Health. 2024 Mar 1;22(1):17. | 2013-2022 | Global | - Aimed to identify factors influencing the retention of Asian IENs - Five key themes: career prospects, downward mobility, inequality in advancement, acculturation, and support systems - The impact of systemic and structural barriers on long-term retention - The importance of tailored retention strategies to address both professional and cultural integration needs |
| Lanada JA, Culligan K. The experiences of internationally educated nurses who joined the nursing workforce in England. Practice Nursing. 2024 Mar 2;35(3):92-8. | 2002-2022 | UK | - Aimed to understand the experiences of IENs during their first 12–24 months in the NHS in England - Themes of unmet expectations, challenges in professional and social integration, and a quest for belongingness - The need for improved cultural, pastoral, and training support to enhance IENs’ transition and retention in the UK healthcare system |
| Sheehy L, Crawford T, River J. The reported experiences of internationally qualified nurses in aged care: A scoping review. Journal of Advanced Nursing. 2024 Apr;80(4):1299-313. | Jan 2010-July 2022 | Aged care; High income countries | - Aimed to explore the reported experiences of IENs working in aged care settings across high-income countries - Key challenges including stress from migration, miscommunication, racism, and the shock of working in aged care - Under-utilisation of skills, fear of de-skilling, and limited career development opportunities - The impact of aged care–specific stigma and lack of professional recognition on IENs’ experiences |
| Kurup C, Betihavas V, Burston A, Jacob E. Strategies employed by developed countries to facilitate the transition of internationally qualified nurses specialty skills into clinical practice: An integrative review. Nursing open. 2023 Dec;10(12):7528-43. | Until August 2023 | Developed countries | - Aimed to review strategies used by developed countries to support the transition of IENs with specialist skills into clinical practice - Most guidelines provided only generic advice on becoming a nurse specialist - Lack of clarity on whether international specialist qualifications are recognised during registration - Ongoing confusion around postgraduate education requirements for specialisation pathways |
| Zulfiqar SH, Ryan N, Berkery E, Odonnell C, Purtil H, O’Malley B. Talent management of international nurses in healthcare settings: A systematic review. Plos one. 2023 Nov 6;18(11):e0293828. | 2012-2022 | Global | - Aimed to synthesise literature on talent management practices for IENs across global healthcare settings - Key challenges across recruitment, retention, and limited career progression opportunities - The need for ongoing professional development tailored to IENs’ needs - Persistent issues of discrimination, cultural barriers, and communication difficulties in the workplace |
| Dahl K, Nortvedt L, Schrøder J, Bjørnnes AK. Internationally educated nurses and resilience: A systematic literature review. International nursing review. 2022 Sep;69(3):405-15. | 2005-2000 | Global | - Aimed to explore how resilience manifests in IENs and what protective factors support them in host countries - Cultural barriers, discrimination, and lack of professional recognition as key challenges to resilience - Personal determination, support systems, and continuous learning were key resilience-enhancing factors - Resilience as shaped by both individual traits and workplace context |
| Balante J, van den Broek D, White K. How does culture influence work experience in a foreign country? An umbrella review of the cultural challenges faced by internationally educated nurses. International Journal of Nursing Studies. 2021 Jun 1;118:103930. | 2000-2019 | Global | - Aimed to synthesise cultural challenges faced by IENs working in foreign countries through an umbrella review - Cultural differences led to feelings of exclusion and outsider status in the workplace - Intercultural communication and differing nursing cultures as major barriers to adaptation - Highlighted how ethnic identity complicated professional adjustment and work integration |
| Abuliezi R, Kondo A, Qian HL. The experiences of foreign‐educated nurses in Japan: a systematic review. International Nursing Review. 2021 Mar;68(1):99-107. | 2013-2020 | Japan | - Aimed to examine the experiences of IENs working in Japan - Identified major challenges including language barriers, failure in national board exams, and adapting to work and social environments - Psychological distress linked to isolation, cultural adjustment, and professional pressure - The need for language support and culturally responsive transition programmes |
| Balante J, van den Broek D, White K. Mixed‐methods systematic review: Cultural attitudes, beliefs and practices of internationally educated nurses towards end‐of‐life care in the context of cancer. Journal of Advanced Nursing. 2021 Sep;77(9):3618-29. | Until March 2020 | End of life care; Global | - Aimed to explore how IENs’ cultural beliefs influence their end-of-life care practices in cancer settings - Cultural background shaped perceptions of patient autonomy and family involvement - Variations in pain management and communication styles due to differing norms - Beliefs about death affected comfort levels and approaches to delivering end-of-life care |
| Bond S, Merriman C, Walthall H. The experiences of international nurses and midwives transitioning to work in the UK: A qualitative synthesis of the literature from 2010 to 2019. International Journal of Nursing Studies. 2020 Oct 1;110:103693. | 2010-2019 | UK | - Aimed to synthesise qualitative research on IENs and midwives’ transition experiences in the UK from 2010 to 2019 - Four main themes: cultural integration, individual challenges, support networks, and communication issues - Difficulties in adapting to UK life and nursing roles, including experiences of discrimination and undervaluation of skills - The need for effective support to facilitate successful integration into work and society |
| Ghazal LV, Ma C, Djukic M, Squires A. Transition-to-US practice experiences of internationally educated nurses: an integrative review. Western Journal of Nursing Research. 2020 May;42(5):373-92. | 2000-2018 | US | - Aimed to analyse and synthesize evidence on transition-to-practice experiences of IENs in the United States - Facilitators include support from family and colleagues, and self-efficacy perceptions - Barriers include stigma related to educational background, communication challenges, cultural differences, practice variations, and legal issues - The need for tailored transition programs to address these challenges and support IENs’ integration into U.S. healthcare settings |
| Davda LS, Gallagher JE, Radford DR. Migration motives and integration of international human resources of health in the United Kingdom: systematic review and meta-synthesis of qualitative studies using framework analysis. Human resources for health. 2018 Dec;16:1-3. | 2010-2020 | UK | - Aimed to examine migration motives and integration experiences of international dental graduates compared to nurses and doctors in the UK - Active recruitment, postgraduate training, and financial gain as common drivers for migration - Integration experiences varied, with nurses reporting wider knowledge gaps, more discrimination, and fewer career progression opportunities than doctors - A lack of research on international dentists’ integration experiences in the UK |
| Lin YQ, Ding Y, Li JY. A literature review of research exploring the experiences of overseas nurses in the United Kingdom (2002–2017). Frontiers of Nursing. 2018;5(1):17-30. | 2002-2017 | UK | - Aimed to critically explore challenges and support mechanisms for IENs working in the UK between 2002 and 2017 - Identified four main challenges: cultural differences, communication issues, unequal opportunities, and de-skilling - Negative impacts on emotions and career development due to these challenges |
| Pung LX, Goh YS. Challenges faced by international nurses when migrating: an integrative literature review. International Nursing Review. 2017 Mar;64(1):146-65. | 2005-2015 | Global | - Aimed to identify challenges faced by IENs post-migration - Major themes: orientation difficulties, longing for familiar practices, professional devaluation, communication barriers, discrimination, personal and professional differences, and need for supportive systems - Emphasized the necessity for multifaceted transition programs and culturally sensitive support to aid adaptation - Concluded that effective integration requires efforts from both international and native nurses |
| Moyce S, Lash R, de Leon Siantz ML. Migration experiences of foreign educated nurses: a systematic review of the literature. Journal of transcultural nursing. 2016 Mar;27(2):181-8. | Until 2013 | Global | - Aimed to synthesise literature on the migration and acculturation experiences of IENs - Common challenges including licensing barriers, language and communication issues, and workplace discrimination - Underutilisation of skills and lack of support impacted professional integration and retention - The role of family and community networks in supporting adaptation and well-being |
| Jenkins BL, Huntington A. A missing piece of the workforce puzzle. The experiences of internationally qualified nurses in New Zealand: A literature review. Contemporary nurse. 2015 Nov 2;51(2-3):220-31. | 2001-2014 | New Zealand | - Aimed to analyse literature on the experiences of IENs transitioning to work in New Zealand, particularly those from the Philippines and India. - Significant challenges faced by IENs during their transition, including cultural differences, communication barriers, and professional integration issues. - Highlighted the scarcity of research focused on the transition experiences of IENs in New Zealand, indicating a need for further investigation into this area. |
| Nichols J, Campbell J. The experiences of internationally recruited nurses in the UK (1995–2007): an integrative review. Journal of Clinical Nursing. 2010 Oct;19(19‐20):2814-23. | 1995-2007 | UK | - Aimed to explore the experiences of IENs in the UK between 1995 and 2007 - Five main themes: motivation for migration, adapting to British nursing, experiences of first-world healthcare, feelings of devaluation and deskilling, and experiences of racial discrimination - Many IENs felt personally and professionally undervalued, leading to emotions of disappointment and unmet expectations - Highlighted implications for job satisfaction and retention, emphasizing the need to address these challenges to prevent staffing shortages in UK healthcare |
| Zizzo KA, Xu Y. Post-hire transitional programs for international nurses: A systematic review. The Journal of Continuing Education in Nursing. 2009 Feb 1;40(2):57-64. | Until Dec 2007 | Global | - Aimed to evaluate the status of post-hire transitional programs for IENs - Reviewed 20 programs, noting that most were not evidence-based - A lack of research on the effectiveness of these programs - Recommended the development of evidence-based programs to facilitate successful transitions |
| Kawi J, Xu Y. Facilitators and barriers to adjustment of international nurses: an integrative review. International nursing review. 2009 Jun;56(2):174-83. | Until Sept 2007 | Global | - Aimed to identify facilitators and barriers encountered by IENs adjusting to foreign healthcare environments. - Facilitators include positive work ethic, persistence, psychosocial and logistical support, assertiveness, and continuous learning. - Barriers include language and communication difficulties, cultural differences, lack of support, inadequate orientation, differences in nursing practice, and experiences of inequality |
| Likupe G. Experiences of African nurses in the UK National Health Service: a literature review. Journal of clinical nursing. 2006 Oct;15(10):1213-20. | Until 2006 | UK | - Aimed to highlight the experiences of Black African nurses working in the UK NHS) - Identified a scarcity of research focused specifically on African nurses, with existing studies concentrating more broadly on internationally recruited or ethnic minority nurses. - Many foreign nurses, including those from Africa, reported negative experiences such as discrimination in pay and working conditions, and exploitation by management. - Raised ethical concerns regarding the recruitment of nurses from developing countries and their subsequent treatment within the UK healthcare system. |
