# Supplementary material for: Experiences of EU and non-EU internationally educated nurses and midwives in the UK: a scoping review
Source: BMC Nurs. 2025 Dec 2;24:1459. doi: 10.1186/s12912-025-04080-y (PMC12673689; doi:10.1186/s12912-025-04080-y)
Supplement: Supplementary file 2 — Supplementary Material 2 [file 12912_2025_4080_MOESM2_ESM.docx]

**Additional file 1. Example search strategy from Ovid MEDLINE**

Medline (Ovid MEDLINE® Epub Ahead of Print, In-Process & Other Non-Indexed Citations, Ovid MEDLINE® Daily and Ovid MEDLINE®) 1946 to present

| **#** | **Query** | **Results from 22 Oct 2023** |
| --- | --- | --- |
| 1 | (afghan* or africa* or albania* or algeria* or angola* or antigua* or barbuda* or argentin* or armenia* or aruba* or azerbaijan* or bahrain* or bangladesh* or bengal* or bangal* or barbados* or barbadian* or bajan or bajans or belarus* or belorus* or byelarus* or byelorus* or belize* or benin* or dahomey or bhutan* or bolivia* or bosnia* or herzegovin* or botswan* or batswan* or bechuanaland* or brazil* or brasil* or bulgaria* or burkina* or burkinese* or upper volta* or burundi* or urundi* or cabo verde* or cape verde* or cambodia* or kampuchea* or khmer* or cameroon* or cameroun* or ubangi shari* or chad* or chile* or china* or chinese or colombia* or comoro* or comore* or comorian* or mayotte* or congo* or zaire* or costa rica* or "cote d'ivoir*" or "cote d' ivoir*" or cote divoir* or cote d ivoir* or ivory coast* or ivorian* or croatia* or cuba or cuban or cubans or "cuba's" or cyprus* or cypriot* or czech* or djibouti* or french somaliland* or dominica* or ecuador* or egypt* or united arab republic* or el salvador* or salvadoran* or guinea* or equatoguinea* or eritrea* or estonia* or eswatini* or swaziland* or swazi* or swati* or ethiopia* or fiji* or gabon* or gabonese* or gabonaise* or gambia* or ((georgia or georgian or georgians) not (atlanta or california or florida)) or ghana* or gibraltar* or greece* or greek* or grecian* or grenada* or grenadian* or guam* or guatemala* or guyana* or guiana* or guyanese* or haiti* or hispaniola* or hondura* or hungary* or hungarian* or india* or indonesia* or iran* or iraq* or isle of man* or jamaica* or jordan* or kazakh* or kenya* or karabati* or korea* or kosovo* or kosova* or kyrgyz* or kirgiz* or kirghiz* or laos or lao or laotian* or latvia* or lebanon* or lebanese* or lesotho* or lesothan* or lesothonian* or basutoland* or mosotho* or basotho* or liberia* or libya* or jamahiriya* or lithuania* or macedonia* or madagasca* or malagasy* or malawi* or nyasaland* or malaysia* or malay* federation or maldives* or maldivian* or indian ocean or mali or malian* or "mali's" or malta or maltese* or "malta's" or micronesia* or marshallese* or kiribati* or marshall island* or nauru or nauran or nauruans or "naurian's" or mariana or marianas or palau or paluan* or tuvalu* or mauritania* or mauritan* or mauritius* or mexico* or mexican* or moldova* or moldovia* or mongol* or montenegr* or morocco* or moroccan* or ifni or mozambique* or mozambican* or myanmar* or burma* or burmese or namibia* or nepal* or new caledonia* or netherlands antill* or nicaragua* or niger* or oman or omani or omanis or "oman's" or pakistan* or palestin* or gaza* or west bank* or panama* or paraguay* or peru or peruvian* or "peru's" or philippine* or philipine* or phillipine* or phillippine* or filipino* or filipina* or poland* or polish or pole or poles or portugal* or portuguese or puerto ric* or romania* or russia* or ussr* or soviet* or rwanda* or rwandese or ruanda* or ruandese or samoa* or navigator island* or pacific island* or polynesia* or "sao tome and principe*" or sao tomean* or santomean* or saudi arabia* or saudi or saudis or senegal* or serbia* or seychell* or sierra leone* or slovak* or sloven* or melanesia* or solomon island* or norfolk island* or somali* or sri lanka* or ceylon* or "saint kitts and nevis*" or "st kitts and nevis*" or kittian* or nevisian* or saint lucia* or st lucia* or saint vincent* or st vincent* or vincentian* or grenadine* or sudan* or surinam* or syria* or tajik* or tadjik* or tadzhik* or tanzania* or tanganyika* or thai* or timor leste* or east timor* or timorese* or togo or togoles* or "togo's" or tonga* or trinidad* or tobago* or tunisia* or turkiy* or turkey* or turk or turks or turkish or turkmen* or uganda* or ukrain* or uruguay* or uzbek* or vanuatu* or new hebrides* or venezuela* or vietnam* or viet nam* or yemen* or yugoslav* or zambia* or zimbabwe* or rhodesia* or arab* countr* or middle east* or global south or sahara* or subsahara* or magreb* or maghrib* or west indies* or caribbean* or central america* or latin america* or south america* or central asia* or north asia* or northern asia* or southeastern asia* or south eastern asia* or southeast asia* or south east asia* or west asia* or western asia* or east europe* or eastern europe* or developing countr* or developing nation* or developing population* or developing world or less developed countr* or less developed nation* or less developed world or lesser developed countr* or lesser developed nation* or lesser developed world or under developed countr* or under developed nation* or under developed world or underdeveloped countr* or underdeveloped nation* or underdeveloped world or middle income countr* or middle income nation* or middle income population* or low income countr* or low income nation* or low income population* or lower income countr* or lower income nation* or lower income population* or underserved countr* or underserved nation* or underserved population* or under served population* or under served nation* or under served population* or deprived countr* or deprived population* or high burden countr* or high burden nation* or countdown countr* or countdown nation* or poor countr* or poor nation* or poor population* or poor world or poorer countr* or poorer nation* or poorer population* or poorer world or developing econom* or less developed econom* or underdeveloped econom* or under developed econom* or middle income econom* or low income econom* or lower income econom* or low gdp or low gnp or low gross domestic or low gross national or lower gdp or lower gnp or lower gross domestic or lower gross national or lmic or lmics or third world or lami countr* or transitional countr* or emerging econom* or emerging nation*).ti,ab,hw,kf. | 3,241,091 |
| 2 | Poland/ or "Poland".mp. | 65,496 |
| 3 | "polish".mp. | 21,046 |
| 4 | Ireland/ or "Republic of Ireland".mp. | 20,923 |
| 5 | Irish.mp. | 11,520 |
| 6 | "Romania".mp. or Romania/ | 15,227 |
| 7 | "Romanian*".mp. | 3,747 |
| 8 | "Australia".mp. or Australia/ | 197,998 |
| 9 | "Australian*".mp. or "Australian Aboriginal and Torres Strait Islander Peoples"/ | 91,293 |
| 10 | United States/ or "United States of America".mp. | 985,795 |
| 11 | "USA".mp. | 129,624 |
| 12 | "American*".mp. | 465,110 |
| 13 | "United Arab Emirates".mp. or United Arab Emirates/ | 4,625 |
| 14 | "Emirati*".mp. | 512 |
| 15 | "Saudi Arabia".mp. or Saudi Arabia/ | 31,989 |
| 16 | "Saudi arabian".mp. | 2,204 |
| 17 | "Hong Kong".mp. or Hong Kong/ | 28,707 |
| 18 | "Hong konger*".mp. | 7 |
| 19 | "Transients and Migrants"/ or "migrant*".mp. or "Emigration and Immigration"/ | 50,776 |
| 20 | "oversea*".mp. | 8,580 |
| 21 | "immigrant*".mp. | 36,024 |
| 22 | "foreign".mp. | 127,966 |
| 23 | "international* qualified".mp. | 32 |
| 24 | "international* recruited".mp. | 27 |
| 25 | "nurs*".mp. or Nurses/ | 818,514 |
| 26 | Midwifery/ or Nurse Midwives/ or "midwi*".mp. | 41,402 |
| 27 | Foreign Professional Personnel/ or Nurses, International/ | 2,463 |
| 28 | "United Kingdom".mp. or exp United Kingdom/ | 416,191 |
| 29 | "UK".mp. | 143,080 |
| 30 | "NHS".mp. | 40,574 |
| 31 | State Medicine/ or "National Health Service*".mp. | 73,012 |
| 32 | 1 or 2 or 3 or 4 or 5 or 6 or 7 or 8 or 9 or 10 or 11 or 12 or 13 or 14 or 15 or 16 or 17 or 18 or 19 or 20 or 21 or 22 or 23 or 24 | 4,762,865 |
| 33 | 25 or 26 | 835,812 |
| 34 | 27 or 33 | 836,458 |
| 35 | 28 or 29 or 30 or 31 | 520,303 |
| 36 | 32 and 34 and 35 | 6,803 |
| 37 | limit 36 to (english language and yr="2010 - 2023") | 2,873 |
